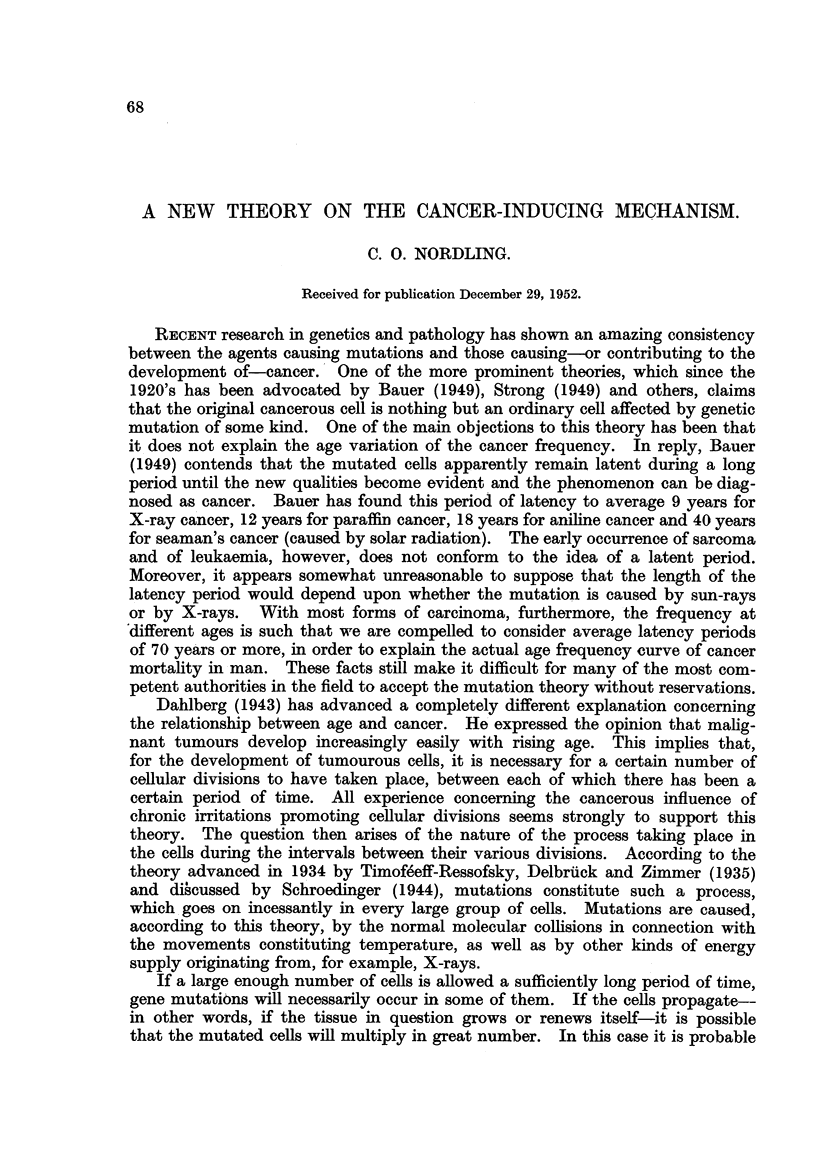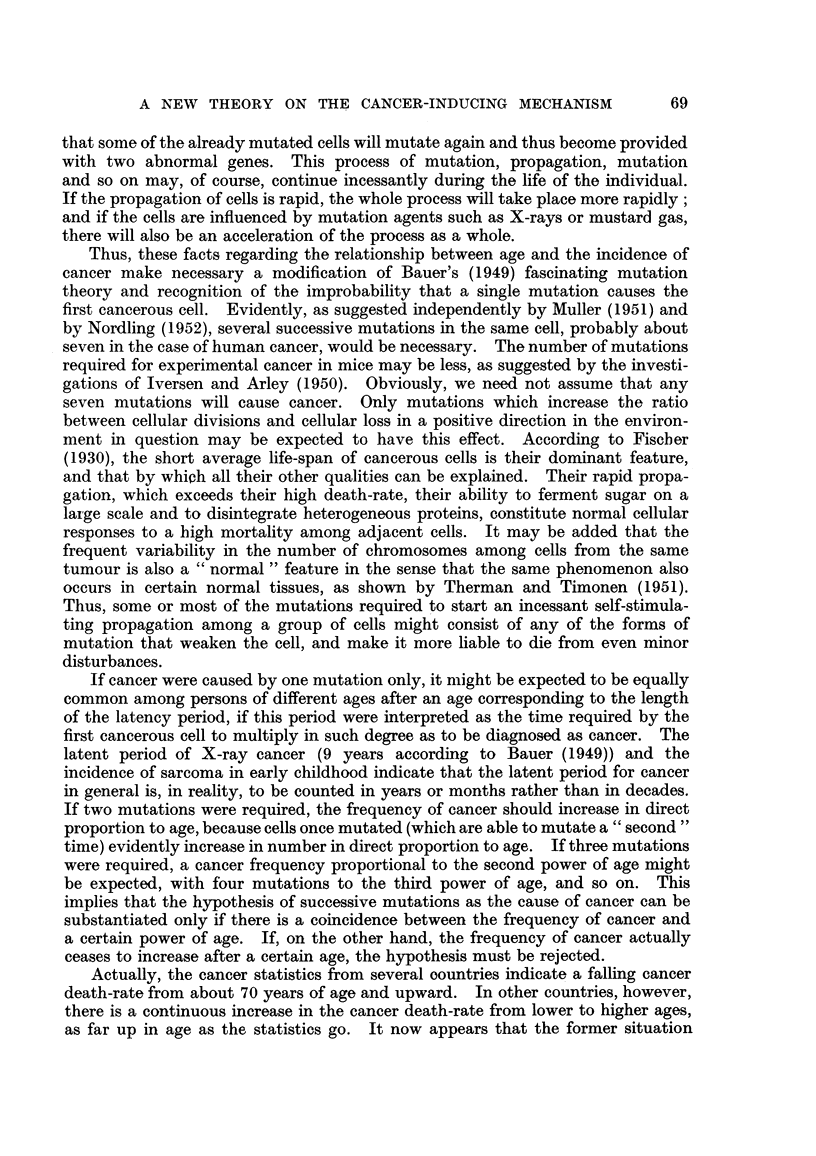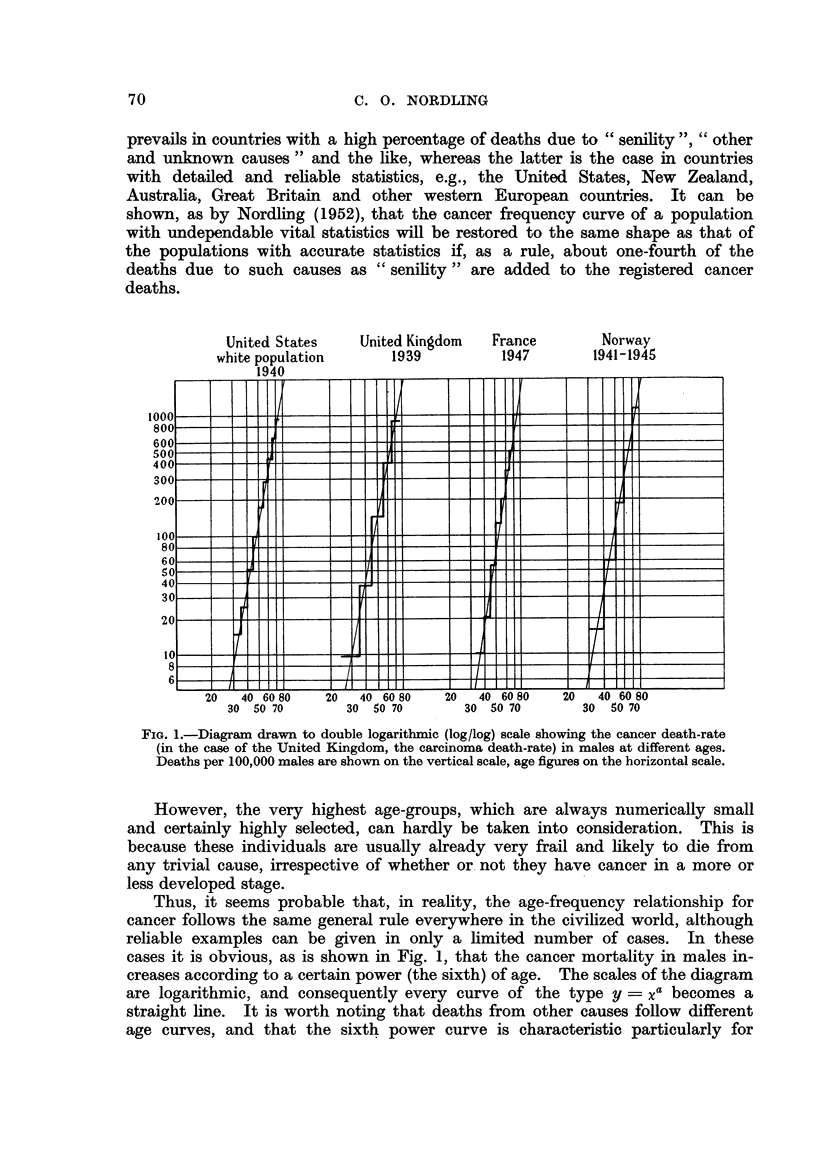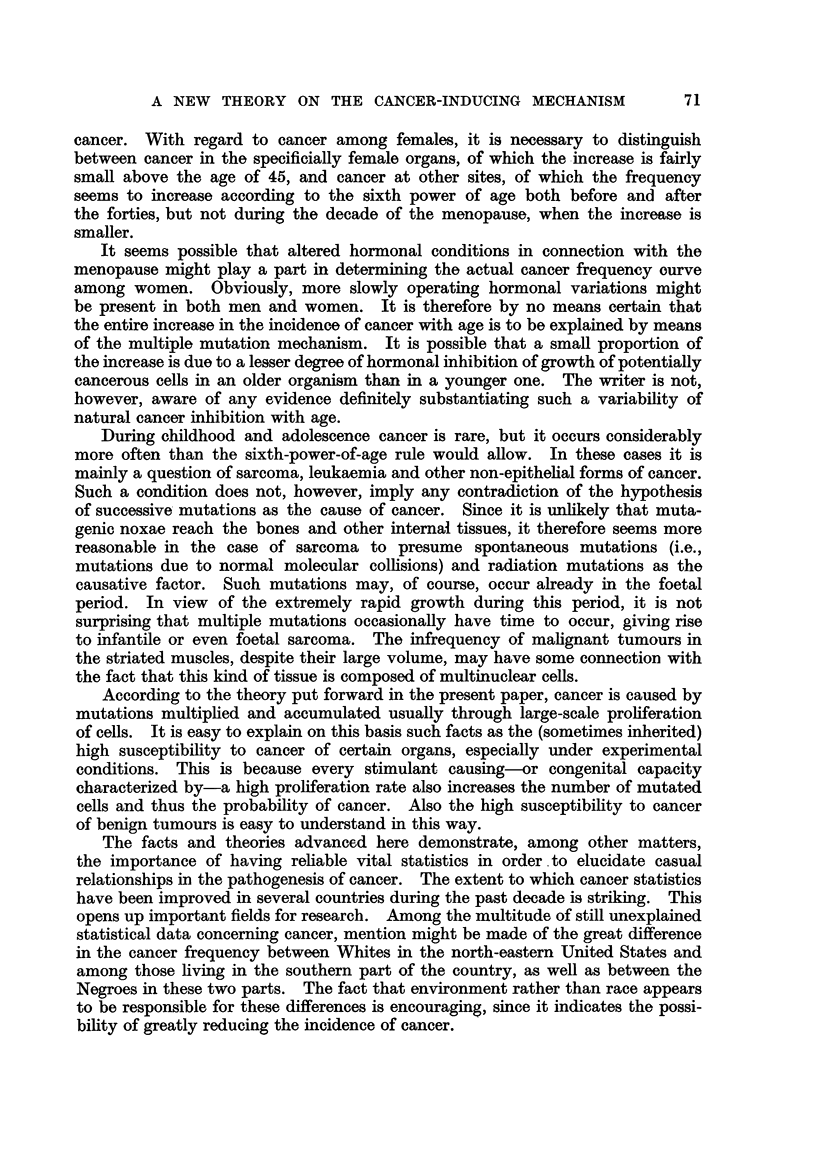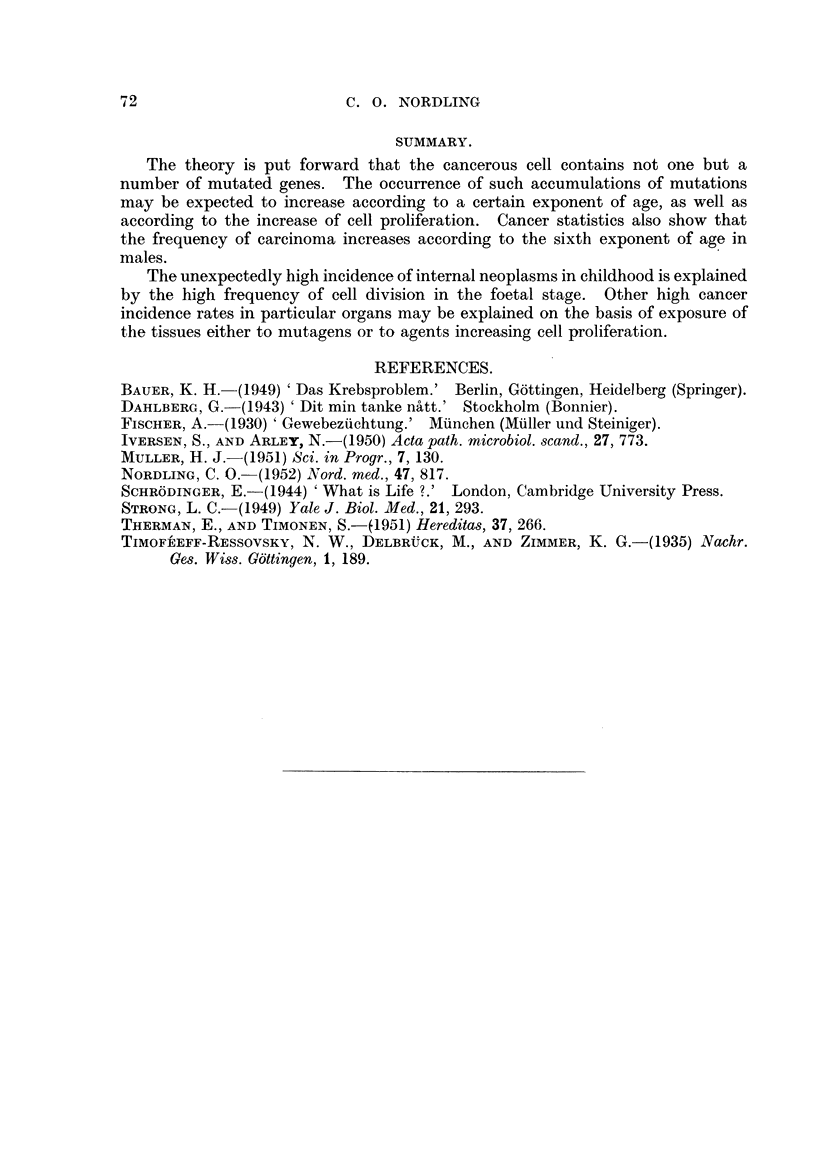# A New Theory on the Cancer-inducing Mechanism

**DOI:** 10.1038/bjc.1953.8

**Published:** 1953-03

**Authors:** C. O. Nordling


### A NEW THEORY ON THE CANCER-INDUCING MECHANISM.

C. O. NORDLING.

Received for publication December 29, 1952.

RECENT research in genetics and pathology has shown an amazing consistency between the agents causing mutations and those causing—or contributing to the development of—cancer. One of the more prominent theories, which since the 1920's has been advocated by Bauer (1949), Strong (1949) and others, claims that the original cancerous cell is nothing but an ordinary cell affected by genetic mutation of some kind. One of the main objections to this theory has been that it does not explain the age variation of the cancer frequency. In reply, Bauer (1949) contends that the mutated cells apparently remain latent during a long period until the new qualities become evident and the phenomenon can be diagnosed as cancer. Bauer has found this period of latency to average 9 years for X-ray cancer, 12 years for paraffin cancer, 18 years for aniline cancer and 40 years for seaman's cancer (caused by solar radiation). The early occurrence of sarcoma and of leukaemia, however, does not conform to the idea of a latent period. Moreover, it appears somewhat unreasonable to suppose that the length of the latency period would depend upon whether the mutation is caused by sun-rays or by X-rays. With most forms of carcinoma, furthermore, the frequency at different ages is such that we are compelled to consider average latency periods of 70 years or more, in order to explain the actual age frequency curve of cancer mortality in man. These facts still make it difficult for many of the most competent authorities in the field to accept the mutation theory without reservations.

Dahlberg (1943) has advanced a completely different explanation concerning the relationship between age and cancer. He expressed the opinion that malignant tumours develop increasingly easily with rising age. This implies that, for the development of tumourous cells, it is necessary for a certain number of cellular divisions to have taken place, between each of which there has been a certain period of time. All experience concerning the cancerous influence of chronic irritations promoting cellular divisions seems strongly to support this theory. The question then arises of the nature of the process taking place in the cells during the intervals between their various divisions. According to the theory advanced in 1934 by Timoféeff-Ressofsky, Delbrück and Zimmer (1935) and discussed by Schroedinger (1944), mutations constitute such a process, which goes on incessantly in every large group of cells. Mutations are caused, according to this theory, by the normal molecular collisions in connection with the movements constituting temperature, as well as by other kinds of energy supply originating from, for example, X-rays.

If a large enough number of cells is allowed a sufficiently long period of time, gene mutations will necessarily occur in some of them. If the cells propagate—in other words, if the tissue in question grows or renews itself—it is possible that the mutated cells will multiply in great number. In this case it is probable that some of the already mutated cells will mutate again and thus become provided with two abnormal genes. This process of mutation, propagation, mutation and so on may, of course, continue incessantly during the life of the individual. If the propagation of cells is rapid, the whole process will take place more rapidly ; and if the cells are influenced by mutation agents such as X-rays or mustard gas, there will also be an acceleration of the process as a whole.

Thus, these facts regarding the relationship between age and the incidence of cancer make necessary a modification of Bauer's (1949) fascinating mutation theory and recognition of the improbability that a single mutation causes the first cancerous cell. Evidently, as suggested independently by Muller (1951) and by Nordling (1952), several successive mutations in the same cell, probably about seven in the case of human cancer, would be necessary. The number of mutations required for experimental cancer in mice may be less, as suggested by the investigations of Iversen and Arley (1950). Obviously, we need not assume that any seven mutations will cause cancer. Only mutations which increase the ratio between cellular divisions and cellular loss in a positive direction in the environment in question may be expected to have this effect. According to Fischer (1930), the short average life-span of cancerous cells is their dominant feature, and that by which all their other qualities can be explained. Their rapid propagation, which exceeds their high death-rate, their ability to ferment sugar on a large scale and to disintegrate heterogeneous proteins, constitute normal cellular responses to a high mortality among adjacent cells. It may be added that the frequent variability in the number of chromosomes among cells from the same tumour is also a " normal " feature in the sense that the same phenomenon also occurs in certain normal tissues, as shown by Therman and Timonen (1951). Thus, some or most of the mutations required to start an incessant self-stimulating propagation among a group of cells might consist of any of the forms of mutation that weaken the cell, and make it more liable to die from even minor disturbances.

If cancer were caused by one mutation only, it might be expected to be equally common among persons of different ages after an age corresponding to the length of the latency period, if this period were interpreted as the time required by the first cancerous cell to multiply in such degree as to be diagnosed as cancer. The latent period of X-ray cancer (9 years according to Bauer (1949)) and the incidence of sarcoma in early childhood indicate that the latent period for cancer in general is, in reality, to be counted in years or months rather than in decades. If two mutations were required, the frequency of cancer should increase in direct proportion to age, because cells once mutated (which are able to mutate a " second " time) evidently increase in number in direct proportion to age. If three mutations were required, a cancer frequency proportional to the second power of age might be expected, with four mutations to the third power of age, and so on. This implies that the hypothesis of successive mutations as the cause of cancer can be substantiated only if there is a coincidence between the frequency of cancer and a certain power of age. If, on the other hand, the frequency of cancer actually ceases to increase after a certain age, the hypothesis must be rejected.

Actually, the cancer statistics from several countries indicate a falling cancer death-rate from about 70 years of age and upward. In other countries, however, there is a continuous increase in the cancer death-rate from lower to higher ages, as far up in age as the statistics go. It now appears that the former situation prevails in countries with a high percentage of deaths due to " senility ", " other and unknown causes " and the like, whereas the latter is the case in countries with detailed and reliable statistics, e.g., the United States, New Zealand, Australia, Great Britain and other western European countries. It can be shown, as by Nordling (1952), that the cancer frequency curve of a population with undependable vital statistics will be restored to the same shape as that of the populations with accurate statistics if, as a rule, about one-fourth of the deaths due to such causes as " senility " are added to the registered cancer deaths.

Fig. 1.—Diagram drawn to double logarithmic (log/log) scale showing the cancer death-rate (in the case of the United Kingdom, the carcinoma death-rate) in males at different ages. Deaths per 100,000 males are shown on the vertical scale, age figures on the horizontal scale.

However, the very highest age-groups, which are always numerically small and certainly highly selected, can hardly be taken into consideration. This is because these individuals are usually already very frail and likely to die from any trivial cause, irrespective of whether or not they have cancer in a more or less developed stage.

Thus, it seems probable that, in reality, the age-frequency relationship for cancer follows the same general rule everywhere in the civilized world, although reliable examples can be given in only a limited number of cases. In these cases it is obvious, as is shown in Fig. 1, that the cancer mortality in males increases according to a certain power (the sixth) of age. The scales of the diagram are logarithmic, and consequently every curve of the type $y = x^a$ becomes a straight line. It is worth noting that deaths from other causes follow different age curves, and that the sixth power curve is characteristic particularly for cancer. With regard to cancer among females, it is necessary to distinguish between cancer in the specificially female organs, of which the increase is fairly small above the age of 45, and cancer at other sites, of which the frequency seems to increase according to the sixth power of age both before and after the forties, but not during the decade of the menopause, when the increase is smaller.

It seems possible that altered hormonal conditions in connection with the menopause might play a part in determining the actual cancer frequency curve among women. Obviously, more slowly operating hormonal variations might be present in both men and women. It is therefore by no means certain that the entire increase in the incidence of cancer with age is to be explained by means of the multiple mutation mechanism. It is possible that a small proportion of the increase is due to a lesser degree of hormonal inhibition of growth of potentially cancerous cells in an older organism than in a younger one. The writer is not, however, aware of any evidence definitely substantiating such a variability of natural cancer inhibition with age.

During childhood and adolescence cancer is rare, but it occurs considerably more often than the sixth-power-of-age rule would allow. In these cases it is mainly a question of sarcoma, leukaemia and other non-epithelial forms of cancer. Such a condition does not, however, imply any contradiction of the hypothesis of successive mutations as the cause of cancer. Since it is unlikely that mutagenic noxae reach the bones and other internal tissues, it therefore seems more reasonable in the case of sarcoma to presume spontaneous mutations (i.e., mutations due to normal molecular collisions) and radiation mutations as the causative factor. Such mutations may, of course, occur already in the foetal period. In view of the extremely rapid growth during this period, it is not surprising that multiple mutations occasionally have time to occur, giving rise to infantile or even foetal sarcoma. The infrequency of malignant tumours in the striated muscles, despite their large volume, may have some connection with the fact that this kind of tissue is composed of multinuclear cells.

According to the theory put forward in the present paper, cancer is caused by mutations multiplied and accumulated usually through large-scale proliferation of cells. It is easy to explain on this basis such facts as the (sometimes inherited) high susceptibility to cancer of certain organs, especially under experimental conditions. This is because every stimulant causing—or congenital capacity characterized by—a high proliferation rate also increases the number of mutated cells and thus the probability of cancer. Also the high susceptibility to cancer of benign tumours is easy to understand in this way.

The facts and theories advanced here demonstrate, among other matters, the importance of having reliable vital statistics in order to elucidate casual relationships in the pathogenesis of cancer. The extent to which cancer statistics have been improved in several countries during the past decade is striking. This opens up important fields for research. Among the multitude of still unexplained statistical data concerning cancer, mention might be made of the great difference in the cancer frequency between Whites in the north-eastern United States and among those living in the southern part of the country, as well as between the Negroes in these two parts. The fact that environment rather than race appears to be responsible for these differences is encouraging, since it indicates the possibility of greatly reducing the incidence of cancer.

SUMMARY.

The theory is put forward that the cancerous cell contains not one but a number of mutated genes. The occurrence of such accumulations of mutations may be expected to increase according to a certain exponent of age, as well as according to the increase of cell proliferation. Cancer statistics also show that the frequency of carcinoma increases according to the sixth exponent of age in males.

The unexpectedly high incidence of internal neoplasms in childhood is explained by the high frequency of cell division in the foetal stage. Other high cancer incidence rates in particular organs may be explained on the basis of exposure of the tissues either to mutagens or to agents increasing cell proliferation.

### REFERENCES.

BAUER, K. H.—(1949) 'Das Krebsproblem.' Berlin, Göttingen, Heidelberg (Springer).
DAHLBERG, G.—(1943) 'Dit min tanke nått.' Stockholm (Bonnier).
FISCHER, A.—(1930) 'Gewebezüchtung.' München (Müller und Steiniger).
IVERSEN, S., AND ARLEY, N.—(1950) *Acta path. microbiol. scand.*, **27**, 773.
MULLER, H. J.—(1951) *Sci. in Progr.*, **7**, 130.
NORDLING, C. O.—(1952) *Nord. med.*, **47**, 817.
SCHRÖDINGER, E.—(1944) 'What is Life ?.' London, Cambridge University Press.
STRONG, L. C.—(1949) *Yale J. Biol. Med.*, **21**, 293.
THERMAN, E., AND TIMONEN, S.—(1951) *Hereditas*, **37**, 266.
TIMOFÉEFF-RESSOVSKY, N. W., DELBRÜCK, M., AND ZIMMER, K. G.—(1935) *Nachr. Ges. Wiss. Göttingen*, **1**, 189.